# Two-dimensional multifractal detrended fluctuation analysis for plant identification

**DOI:** 10.1186/s13007-015-0049-7

**Published:** 2015-02-26

**Authors:** Fang Wang, Deng-wen Liao, Jin-wei Li, Gui-ping Liao

**Affiliations:** College of Science, Hunan Agricultural University, Changsha, 410128 China; Forestry Department of Hunan Province, Quality Testing and Inspection Centre of Forest Products, Changsha, 410007 China; Agricultural Information Institute, Hunan Agricultural University, Changsha, 410128 China

**Keywords:** Plant identification, Multifractal detrended fluctuation analysis, Support vector machines and kernel methods

## Abstract

**Background:**

In this paper, a novel method is proposed to identify plant species by using the two- dimensional multifractal detrended fluctuation analysis (2D MF-DFA). Our method involves calculating a set of multifractal parameters that characterize the texture features of each plant leaf image. An index, *I*_0_, that characterizes the relation of the intra-species variances and inter-species variances is introduced. This index is used to select three multifractal parameters for the identification process. The procedure is applied to the Swedish leaf data set containing leaves from fifteen different tree species.

**Results:**

The chosen three parameters form a three-dimensional space in which the samples from the same species can be clustered together and be separated from other species. Support vector machines and kernel methods are employed to assess the identification accuracy. The resulting averaged discriminant accuracy reaches 98.4% for every two species by the 10 − fold cross validation, while the accuracy reaches 93.96% for all fifteen species.

**Conclusions:**

Our method, based on the 2D MF-DFA, provides a feasible and efficient procedure to identify plant species.

## Introduction

The increasing interest in biodiversity and biocomplexity, together with the growing availability of digital images and image analysis algorithms, makes plant species identification and classification a topic that has attracted many researchers’ attention. In general, many parts of a plant such as flowers, seeds, roots, and leaves can be used to identify plant species [1-3]. In this paper, we focus on the usage of image of leaves as they are widely available. Leaf’s shape, color, vein properties, texture and contours are important features for plant identification. For example, leaf shapes were used in [4-6]; complex veins and contours of leaves were used in [7] and leaf texture was used in [8-11] for plant species identification. For plant species identification using digital morphometrics, we refer the reader to [12-14] and the references therein.

Note that in [7], a monofractal method was used to extract plant leaf’s features from leaf images. This method was then used in [15,16]. It’s been recognized that the monofractal method cannot fully extract detailed information from the leaf image and therefore cannot be efficiently applied to process the images of the objects that are locally irregular [17]. To overcome this difficulty, several multifractal analysis (MFA) methods were proposed [18-22]. For example, Backes *et al.* [18,19] used multi-scale fractal dimensions to describe the texture property of leaf’s surface to identify plants, which turned out to be very efficient. Note that the classical MFA is based on capacity measurement or probability measurement and thus describes only stationary measurements [17]. For a leaf image, the surface itself is hardly stationary. Therefore, the multifractal detrended fluctuation analysis (MF-DFA) method that can deal with non-stationary is a desirable method for leaf image analysis [23]. Though the MF-DFA method has been successfully applied in many fields for non-stationary series and surfaces [24-30], to the best of our knowledge, no work yet has applied the MF-DFA on leaf images for plant identification and classification. In this paper, we attempt to identify plant species *via* leaf images by using the MF-DFA. More precisely, we first adopt the MF-DFA to extract important texture features from leaf images and obtain several key multifractal parameters, and then we apply the support vector machines and kernel methods (SVMKM) to distinguish leaves from different plant species. The widely used Swedish leaf data set [31] containing leaves from fifteen different Swedish tree species are used for our experiments. Our results show that the average accuracy is 98.4% for every two species by the 10 − fold cross validation; for the over-all species, the average accuracy reaches 93.96% by the same validation criterion.

We organize the rest of this paper as follows: in Methods and materials we adopt the two-dimensional (2D) MF-DFA to calculate the multifractal parameters. In Results and discussion, we present and discuss our results. Our method is then further tested in Model test. A summary is provided in Conclusions.

## Methods and materials

### Multifractal detrended fluctuation analysis

We first adopt the 2D MF-DFA method proposed in [32] to our setting as follows:

- Step 1: Regard a leaf image as a self-similar surface and represent it by an *M × N* matrix *X* = (*X(i, j*)), *i* = 1, 2,…, *M* and *j* = 1, 2,…, *N*. Partition the surface into *M*_*s*_ *× N*_*s*_ non-overlapping square sub-surface of equal length *s*, where *M*_*s*_ ≡ [*M* / *s*] and *N*_*s*_ ≡ [*N* / *s*] are positive integers (Here [*u*] stands for the largest integer that is less than or equal to *u*). Each sub-surface is denoted by *X*_*m,n*_ *= X*_*m,n*_(*i*, *j*) with *X*_*m,n*_(*i*, *j*) = *X*(*r* + *i*, *t* + *j*) for 1 ≤ *i*, *j* ≤ *s*, where *r* = (*m*-1)*s* and *t* = (*n*-1)*s*. Note that *M* and *N* are not necessarily multiples of the length s, therefore, the sub-surfaces in the upper-right and the bottom may not be taken into consideration. We can then repeat the partitioning procedure starting from the other three corners.
- Step 2: For each sub-domain *X*_*m,n*_, find its cumulative sum
  1 $$ {G}_{m,n}\left(i,j\right)={\displaystyle \sum_{k_1=1}^i{\displaystyle {\sum_{k_2=1}^j}_{m,n}Xm\left({k}_1,{k}_2\right)}}, $$
- where 1 ≤ *i*, *j* ≤ *s*, *m* = 1, 2, …, *M*_*s*_ and *n* = 1, 2, …, *N*_*s*_. Then *G*_*m*,*n*_ = *G*_*m*,*n*_(*i*, *j*) (*i*, *j* = 1, 2, · · ·, *s*) itself is a surface.
- Step 3: For each surface *G*_*m*,*n*_, obtain a local trend *G*^~^_*m*,*n*_ by fitting it with a pre-chosen bivariate polynomial function. In this paper, we choose the trending function as
  2 $$ {\tilde{G}}_{m,n}\left(i,j\right)= ai+bj+c, $$
- a.where 1 ≤ *i*, *j* ≤ *s* and *a*, *b* and *c* are free parameters to be determined by the least-squares method. The residual matrix is then given by *y*_*m*,*n*_ = *y*_*m*,*n*_(*i*, *j*) with
  3 $$ {y}_{m,n}\left(i,j\right)={G}_{m,n}\left(i,j\right)-{\tilde{G}}_{m,n}\left(i,j\right). $$
- Step 4: Define the detrended fluctuation function *F*(*m*, *n*, *s*) for the segment *X*_*m,n*_ as follows:
  4 $$ {F}^2\left(m,n,s\right)=\frac{1}{s^2}{\displaystyle \sum_{i=1}^s{\displaystyle \sum_{j=1}^s{y}_{m,n}{\left(i,j\right)}^2}} $$
- and the *q*th-order fluctuation function
  5 $$ {F}_q(s)={\left[\frac{1}{M_s{N}_s}{\displaystyle \sum_{m=1}^{M_s}{\displaystyle \sum_{n=1}^{N_s}{\left[F\left(m,n,s\right)\right]}^q}}\right]}^{1/q},q\ne 0. $$
  6 $$ {F}_q(s)= \exp \left\{\frac{1}{M_s{N}_s}{\displaystyle \sum_{m=1}^{M_s}{\displaystyle \sum_{n=1}^{N_s} \ln \left[F\left(m,n,s\right)\right]}}\right\},\ q=0. $$
- Step 5: Vary the value of *s* ranging from 6 to min(*M*, *N*)/4. If there is long-range power-law correlation for large values of s, then
  $$ {F}_q(s)\propto {s}^{h(q)}. $$

This allows us to obtain the scaling exponent *h*(*q*) *via* linearly regressing ln*F*_*q*_(*s*) on ln*s*. Note that *h*(2) is the so called Hurst index of the surface, we then call *h*(*q*) the generalized Hurst index of the surface. For each *q*, the corresponding classical multifractal scaling exponent *τ*(*q*) is given by:

7 $$ \tau (q)=qh(q)-{D}_f=qh(q)-2, $$

where *D*_*f*_ is the fractal dimension of the geometric support of the multifractal measure, and takes the value of *D*_*f*_ = 2 in our work. The generalized multifractal dimension *D*_*q*_ is then given by

8 $$ {D}_q=\frac{\tau (q)}{q-1}=\frac{qh(q)-2}{q-1},\ q\ne 1. $$

In the case where *q* = 1, *D*_1_ can be obtained *via* a linear regression of ${\displaystyle {\sum}_{m=1}^{M_s}{\displaystyle {\sum}_{n=1}^{N_s}{P}_{m,n} \ln {P}_{m;n}}}$ against ln*s*, where

$$ {P}_{m,n}=\frac{{\displaystyle {\sum}_{1\le i,j\le s}{X}_{m,n}\left(i,j\right)}}{{\displaystyle {\sum}_{1\le i\le M}{\displaystyle {\sum}_{1\le j\le N}X\left(i,j\right)}}}. $$

The other two indicators characterizing the singularity strength of the multifractal surface are the *Hölder* exponent *α*(*q*) and the singularity spectrum *f* (*α*), which are given by

9 $$ \alpha (q)={\tau}^{\prime }(q)=h(q)+q{h}^{\prime }(q),\kern0.5em f\left(\alpha \right)=q\alpha (q)-\tau (q)=q\left[\alpha -h(q)\right]+2. $$

Here *α*(*q*) characterizes the local singularity of an image texture, and *f* (*α*) measures the global singularity of an image texture. Varying the value of *q* in the range from −15 to 15 determines ∆*α* and ∆*f* as follows:

10 $$ \Delta \alpha ={\alpha}_{\max }-{\alpha}_{\min },\Delta f=f\left({\alpha}_{\max}\right)-f\left({\alpha}_{\min}\right), $$

*α*_max_ = max{*α*(*q*), *q*∈[−15,15]} and *α*_min_ = min{*α*(*q*), *q*∈[−15,15]}. Note that the index ∆*α* is considered as an indicator to measure the absolute magnitude of the gray scale volatility. The larger value of ∆*α*, the smaller even distribution of probability measure and the more roughness image surface will be expected. The index ∆*f* is the *Hausdorff* dimension of the measure object, which measures the degree of confusion. Therefore both ∆*α* and ∆*f* are important multifractal parameters in describing the characteristics of an image in our study.

### Experiment materials

**Figure 1:**
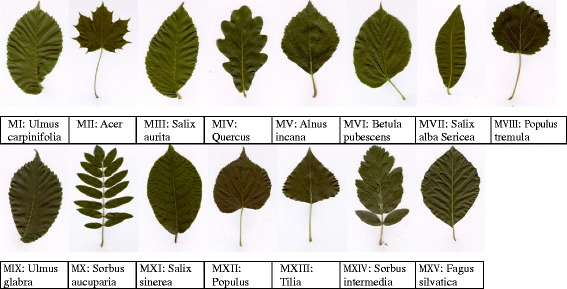
**Fifteen species of tree leaf images from the Swedish leaf database, their species’ name and corresponding.**

To demonstrate our method of identifying plant species by using the leaf texture, we use the Swedish leaf data set [31] for our experiment, which is widely employed in computer vision and pattern recognition fields [4,33,34], plant taxon fields [1] and image processing fields [6,35]. This leaf data set has images of 15 species of leaves with 75 sample images per species. We label the fifteen species by MI, MII, · · ·, MXV (See Figure 1).

We first transform the color image to gray scale so that each image can be viewed as a three- dimensional surface with the first two coordinates (*i*, *j*) denoting the 2D position and the third coordinate *z* denoting the gray level of the corresponding pixel.

### Multifractal nature of image surfaces

Each image is stored as a 2D matrix in 256 grey levels. This allows us to follow the procedure introduced in Multifractal detrended fluctuation analysis to calculate the associated *h*(*q*) and *τ*(*q*). If *τ*(*q*) is nonlinear in *q*, that is *h*(*q*) is not independent of *q*, then the image possesses the multifractal nature.

**Figure 2:**
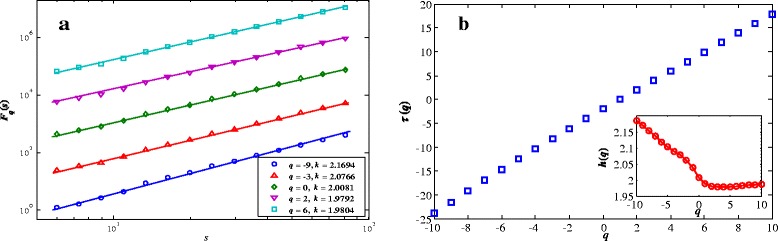
**Multifractal nature in the power-law of the gray image of leaf MIV004. (a)**: The plots of the detrended fluctuation function *F*_*q*_(*s*) for different values of *q*. In order to make clearer contrast among the different curves, some constants are subtracted. The straight lines are the best fitted lines whose slopes are shown in the legend. **(b)**: Dependence of *τ*(*q*) and *h*(*q*) on *q*.

**Figure 3:**
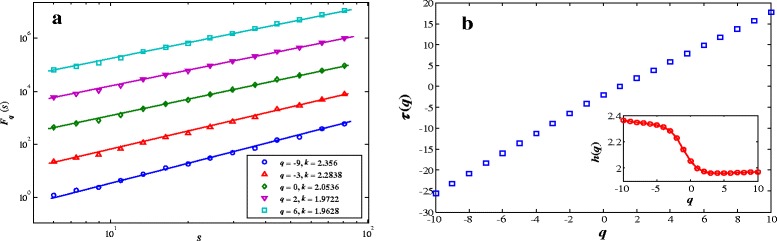
**Multifractal nature in the power-law of the gray image of leaf MX017. (a)**: The plots of the detrended fluctuation function *F*_*q*_(*s*) for different values of *q*. In order to make clearer contrast among the different curves, some constants are subtracted. The straight lines are the best fitted lines whose slopes are shown in the legend. **(b)**: Dependence of *τ*(*q*) and *h*(*q*) on *q*.

For the Swedish leaf data set, we find that the leaf images all possess the multifractal nature. Figure 2 and Figure 3 demonstrate the multifractal nature of two randomly chosen leaf images, namely, image MIV004 and image MX017, the former has 1793 × 979 pixels and the latter has 2934 × 1771 pixels. In each the left panel illustrates the dependence of the detrended fluctuation function *F*_*q*_(*s*) as a function of the scale s for different *q*. The well fitted straight lines indicate the evident power law scaling of *F*_*q*_(*s*) versus *s*. The right panel shows that *τ*(*q*) is nonlinear in *q*, indicated by the fact that *h*(*q*) depends on *q*.

## Results and discussion

**Figure 4:**
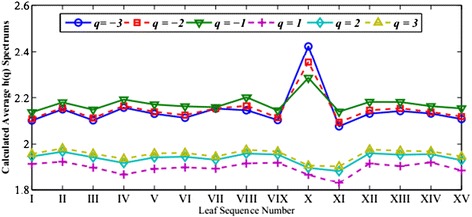
**The generalized Hurst exponents** ***h*** **(** ***q*** **) for each species.**

**Figure 5:**
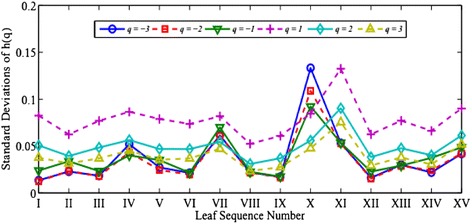
**The standard deviations of the averaged** ***h*** **(** ***q*** **) calculated in Figure** 4 **.**

**Figure 6:**
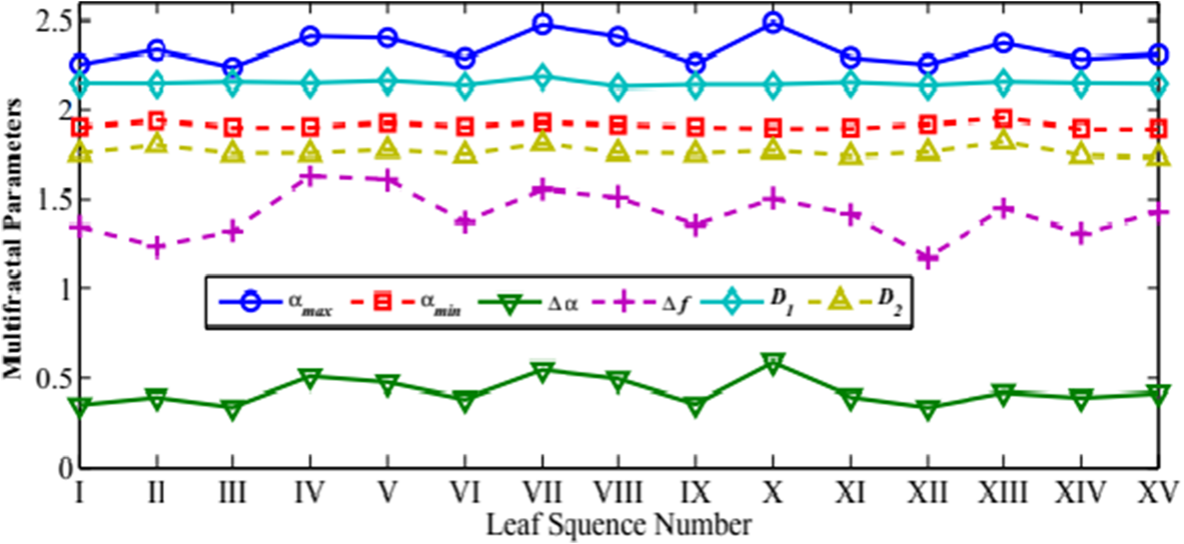
**The averaged values for six related multifractal parameters based the MF-DFA estimation for each species.**

**Figure 7:**
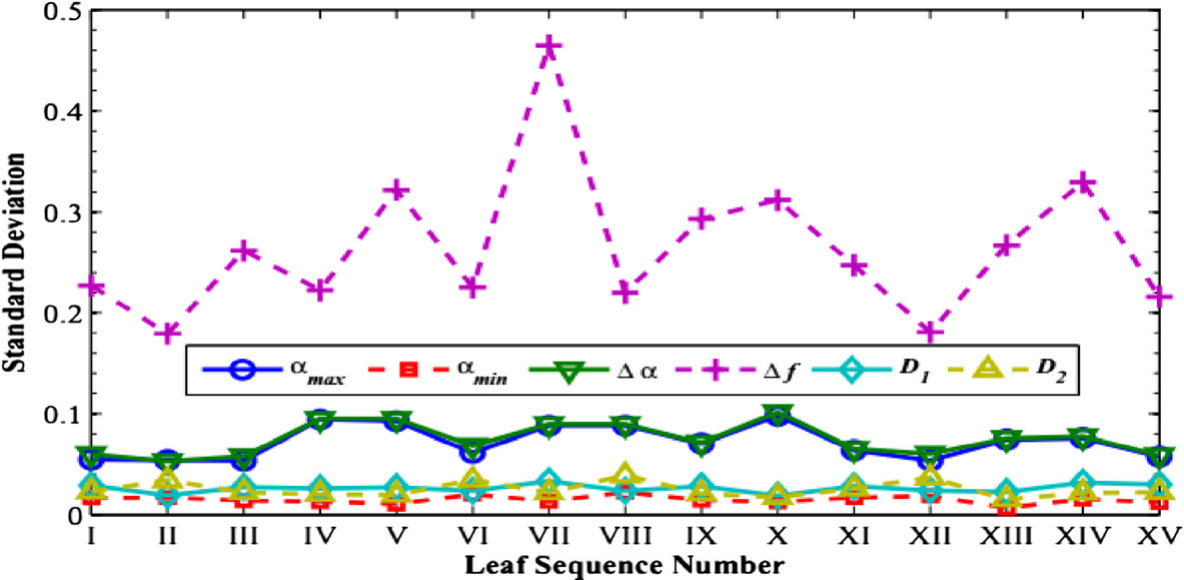
**The standard deviations of the averaged parameter values calculated in Figure** 6 **.**

For each image, we can calculate the generalized Hurst exponents *h*(*q*) and six other multifractal parameters including *α*_max_, *α*_min_, *∆α*, *∆f*, *D*_1_ and *D*_2_. For each tree species, we take the averaged value over the 75 samples and report our calculated values in Figures 4 and 5. Their standard deviations are given in Figures 6 and 7, respectively.

As seen in Figure 4, comparing with *h*(2) and *h*(3), the estimations of *h*(−3), *h*(−2), *h*(−1) and *h*(1) vary in relatively wider dynamic ranges and thus demonstrate better abilities to distinguish textures among different species. Yet, one notes that there are relatively large variations in the standard deviations among the 75 samples for the *h*(*q*) exponents in Figure 5. This suggests that this indicator alone may not be adequate to identify the fifteen tree species. Also as seen in Figure 6 that the three parameters, *α*_max_, ∆*α*, and ∆*f* admit wider dynamic ranges than the other three parameters do. The variations among the 75 samples in the same tree species are notably large as shown in Figure 7.

For species *i* (*i* = I, II, · · ·, XV), with respect to each calculated multifractal parameter, we denote the standard deviation of the 75 samples by *σ*_*in*_(*i*) and define *σ*_*in*_ as

11 $$ {\sigma}_{in}=\frac{1}{15}{\displaystyle \sum_{i=\mathrm{I}}^{\mathrm{XV}}{\sigma}_{in}(i)}, $$

which represents the intra-species variance. Note also that for each indicator, we can calculate its value corresponding to each species and there are 15 values in total for those 15 species. We define *σ*_*bet*._ as the standard deviation of these 15 calculated values. Then the term *σ*_*bet*._ represents the inter-species variance for each multifractal indicator. We now define an index, *I*_0_, as

12 $$ {I}_0 = \frac{\sigma_{bet.}\ }{\sigma_{in}}. $$

From the definition, we note that the multifractal parameter with larger *I*_0_ serves better as an indicator to distinguish species. We present the calculated values of *I*_0_ in Table 1.

**Table 1:** **The calculated** *σ*_*bet*._ **,** *σ*_*in*_ **and** ***I***_0_ **for the 12 multifractal parameters**

**Parameters**	***h*** **(−3)**	***h*** **(−2)**	***h*** **(−1)**	***h*** **(1)**	***h*** **(2)**	***h*** **(3)**	***α***_max_	***α***_min_	***Δα***	***Δf***	***D***_1_	***D***_2_
*σ*_*bet*._	0.0605	0.0403	0.0363	0.0255	0.0237	0.0233	0.0845	0.0183	0.0806	0.1327	0.0140	0.0259
*σ*_*in*_	0.0368	0.0342	0.0381	0.0779	0.0496	0.0395	0.0722	0.0151	0.0646	0.2645	0.0264	0.0252
*I*_0_	**1.6459**	1.1810	0.9548	0.3280	0.4777	0.5891	1.1705	**1.2132**	**1.2469**	0.5015	0.5316	1.0284

Tip: the symbol bold numbers mean the best choice yielding the top three *I*_0_ indices.

We choose the combination of three multifractal parameters with larger *I*_0_ values, namely, {*h*(−3), *α*_min_, *Δα*}, as the feature descriptors for our classification purpose and apply the support vector machines and kernel methods (SVMKM) with the heavy-tailed radial basis function-’*htrfb*’ as the kernel [36]. It is worth mentioning that the combination of 4 or more parameters does not lead to significant higher accuracies, but at a cost with much longer computational time and with no visual advantages. In this sense, the combination of the above three parameters is optimal. For the total sample set containing 75 × 15 = 1125 samples, we use the K − fold cross validation to evaluate the learning performance. This means that 100 (K − 1)/K% samples are randomly chosen as a training set and the remaining 100/K% samples are considered as a test set. The calculation process is then repeated 10 times to eliminate the impact of randomness.

**Figure 8:**
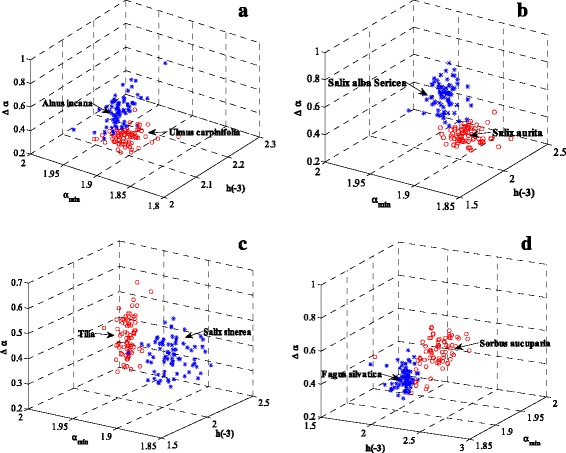
**Visualization of two tree species in the** **{** ***h*** **(−3),** ***α***_min_ **,** ***Δα*** **} space. (a)**: *Ulmuscarpinifolia* versus *Alnusincana*; **(b)**: *Salixaurita* versus *SalixalbaSericea*; **(c)**: *Salixsinerea* versus *Tilia*; **(d**): *Sorbusaucuparia* versus *Fagussilvatica*.

In our first identification experiment, we test the proposed method through examining the distinguishing effect for every two species. To this end, we form a three-dimensional parameter space with components given by the above chosen feature descriptors {*h*(−3), *α*_min_, *Δα*}. In this space, one point represents a leaf sample image. In Figure 8(a)-(d), we plot the corresponding points for *Ulmus carpinifolia* versus *Alnus incana*, *Salix aurita* versus *Salix alba Sericea*, *Salix sinerea* versus *Tilia* and *Sorbus aucuparia* versus *Fagus silvatica*, respectively. As shown in these plots, the samples from the same tree species are clustered together reasonably well.

**Figure 9:**
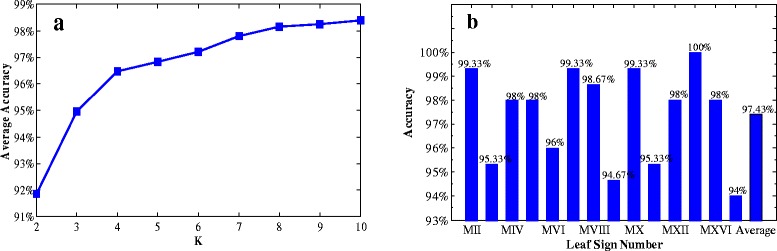
**Average identification accuracies. (a)**: the average accuracies of every two species using different values of K; **(b)**: The accuracies of identifying species *Ulmus carpinifolia* versus the other 14 species using K = 10.

In addition, we calculate the discriminant accuracies of every two tree species by SVMKM using the K − fold cross validation with different K values. The average accuracies of 10 trials are shown in Figure 9(a). To display the applicability of identifying different tree species by our proposed method, as an example, we plot the accuracy of identifying species MI (*Ulmus carpinifolia*) versus other 14 species with K = 10 in Figure 9(b). As expected, the average accuracy of every two species is increasing with respect to K. The obtained best accuracy is 98.40%, higher than 96.82% reported in [35], which requires a very complex pre-processing process for leaf images. It is seen from Figure 9(b) that there are accuracy variations between species *Ulmus carpinifolia* and the other 14 species. Five species, namely, *Salix aurita*, *Betula pubescens*, *Ulmus glabra*, *Salix sinerea* and *Fagus silvatica*, have accuracies below the average accuracy. This suggests that species *Ulmus carpinifolia* has high similarity with the above mentioned five species, which agrees with the observation from Figure 1.

**Figure 10:**
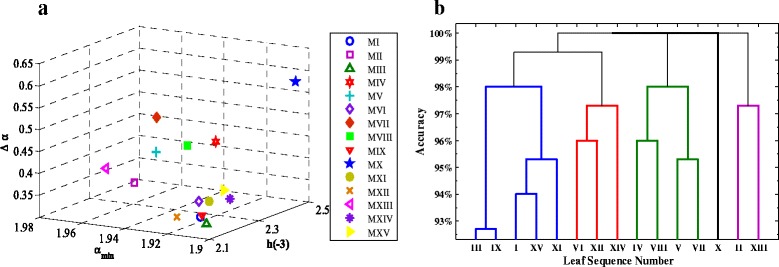
**Feature descriptors of the 15 species and clustering result based on them.** **(a)**: Visualization of averaged indicators over 75 samples in each tree species in the {*h*(−3), *α*_min_, Δ*α*} space; **(b)**: Clustering analysis result on the 15 tree species.

For each species, the averaged {*h*(−3), *α*_min_, *Δα*} of the 75 samples is represented by a single point in the three-dimensional parameter space (see Figure 10) in which different points representing different species may be clustered into several groups. We use the calculated discriminant accuracy of every two species as the distance between these two points (species). This allows us to conduct a cluster analysis for all samples of the 15 species by the method of hierarchical clustering [37]. The result is given in Figure 10(b), which suggests that the 15 tree species’ leaf samples can be clustered into five groups: (i) {*Ulmus carpinifolia*, *Salix aurita*, *Ulmus glabra*, *Salix sinerea*, *Fagus silvatica*}; (ii) {*Betula pubescens*, *Populus*, *Sorbus intermedia*}; (iii) {*Quercus*, *Alnus incana*, *Salix alba Sericea*, *Populus tremula*}; (iv) {*Acer*, *Tilia*} and (v) {*Sorbus aucuparia*}. This is consistent with visualizing the images directly from Figure 1 showing our proposed approach is applicable.

**Figure 11:**
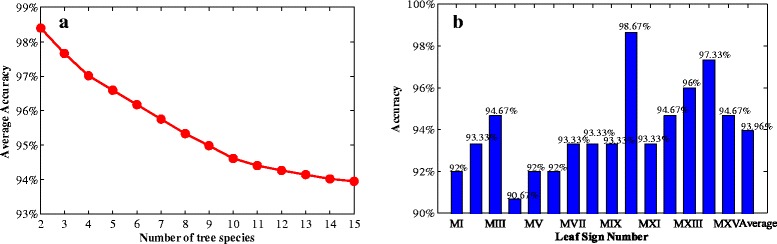
**Identification accuracies of the 15 species calculated with K = 10. (a)**: The average accuracies with respect to different numbers of tree species; **(b)**: The accuracies of the 15 species.

As another important aspect of identification experiment, we next test our method through calculating the identification accuracies for different numbers of species. The averaged accuracy result calculated when K = 10 is shown in Figure 11(a). Note that the average accuracy is decreasing as the number of tree species increases. This is due to the increasing probability of incorrect classification. However, under the worst situation, all 75 × 15 = 1125 sample leaf images are well mixed together, which gives the lowest average accuracy: 93.96%. This is still very convincing that our approach is feasible. We calculate the identification accuracy also when K = 10 for each species and report the result in Figure 11(b), while the identification result for each species is displayed in Table 2. The best three accuracies reach 98.67%, 97.33% and 96%, and the corresponding species are *Sorbus aucuparia*, *Sorbus intermedia* and *Tilia*. As is seen in Figure 1, these three species are clearly distinct from the other species in leaf shapes and textures. This again shows that our method is effective and feasible.

**Table 2:** **The results of identification for the fifteen species of tree leaves by the method of SVMKM with K = 10**

	**MI**	**MII**	**MIII**	**MIV**	**MV**	**MVI**	**MVII**	**MVIII**	**MIX**	**MX**	**MXI**	**MXII**	**MXIII**	**MXIV**	**MXV**
MI	69	0	2	0	1	0	0	0	3	0	0	0	0	0	0
MII	0	70	0	0	1	0	1	0	1	1	0	1	0	0	0
MIII	1	0	71	0	0	0	0	0	0	0	2	1	0	0	0
MIV	1	0	1	68	1	0	0	3	1	0	0	0	0	0	0
MV	0	2	0	0	69	0	0	2	1	0	0	1	0	0	0
MVI	1	0	1	1	0	69	0	0	1	0	1	1	0	0	0
MVII	0	2	0	1	2	0	70	0	0	0	0	0	0	0	0
MVIII	0	0	0	1	2	0	1	70	0	0	1	0	0	0	0
MIX	1	0	0	0	1	1	0	0	70	0	1	1	0	0	0
MX	0	0	0	0	0	0	0	0	0	74	1	0	0	0	0
MXI	1	0	1	0	0	0	0	0	3	0	70	0	0	0	0
MXII	0	0	1	1	0	1	1	0	0	0	0	71	0	0	0
MXIII	0	0	0	0	0	0	0	0	0	0	0	0	72	0	3
MXIV	0	0	0	0	0	0	0	0	0	0	0	0	0	73	2
MXV	0	0	0	0	0	0	0	0	0	0	0	0	2	2	71

**Figure 12:**
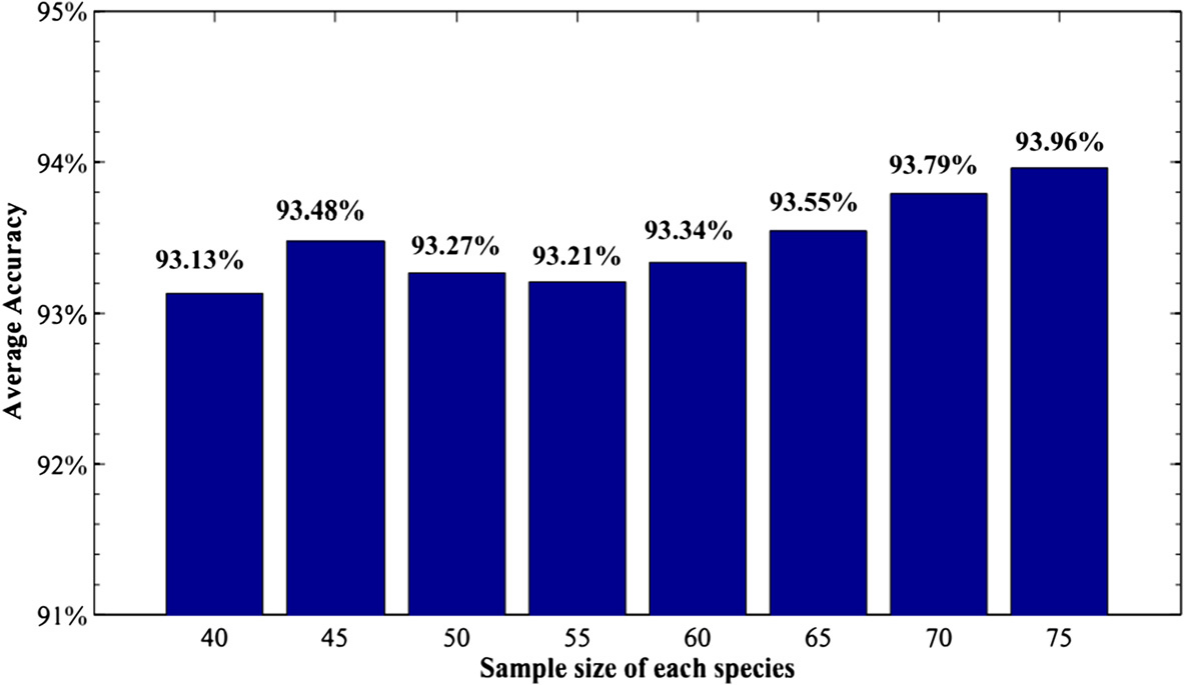
**Average identification accuracies of the 15 species calculated with K = 10 with different species sample sizes.**

We remark that the sample size of each species has little effect on the average discriminant accuracy. To justify this, we randomly choose *n* (*n* ≤ 75) leaf samples for each species and run the procedure. Then repeat the process 10 times and take the average accuracy, which is reported in Figure 12. It can be seen from Figure 12 that as the number of samples changes from 40 to 75, the accuracy changes only 0.73%.

## Model test

**Figure 13:**
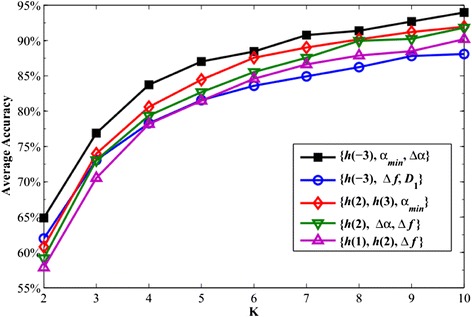
**The average accuracies of the 15 species for the selected combinations with increasing K.**

In this section, we test our proposed method to demonstrate its efficiency. More precisely, we test the validity of the optimal multifractal parameter combination {*h*(−3), *α*_min_, *Δα*}. To this end, we choose other four combinations composed by three multifractal parameters to construct four three-dimensional spaces from Table 1. These four choices are {*h*(−3), *Δf*, *D*_1_}, {*h*(2), *h*(3), *α*_min_}, {*h*(2), *Δα*, *Δf*} and {*h*(1), *h*(2), *Δf*}. One notes that each of the first three combinations contains one multifractal parameter from {*h*(−3), *α*_min_, *Δα*} and the fourth combination consists of the three parameters that produce the three smallest *I*_0_ values. As in the procedure proposed in the previous subsection, we place the 1125 leaf samples into the four new three-dimensional spaces and also use the SVMKM to distinguish them. Under the K − fold cross validation, the discriminant accuracies with increasing K are shown in Figure 13. Obviously, the highest accuracy still comes from the combination {*h*(−3), *α*_min_, *Δα*} for each K and the lowest accuracy comes from the combination {*h*(1), *h*(2), *Δf*}. This again suggests that the index *I*_0_ successfully indicates the optimal multifractal parameter combination.

## Conclusions

**Figure 14:**
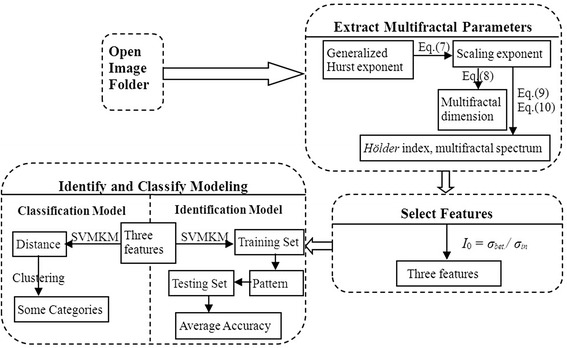
**The flow chart of software programing base on our model is as follows.** Detailed codes are available upon request.

In this paper we have adopted the 2D MF-DFA method proposed in [32] to extract important texture features from leaf images. This allow us to calculate the generalized Hurst exponents, *h*(*q*), and several other multifractal parameters including *α*_max_, *α*_min_, ∆*α*, ∆*f*, *D*_1_ and *D*_2_. By defining an index, *I*_0_, which examines the variation of the inter-species variances and the intra-species variances, we are able to find an optimal combination of the multifractal parameters that best characterizes the key features of plant species allowing high accuracy in plant species identification. For the Swedish leaf data set which contains 15 species and 75 × 15 = 1125 samples in total [31], the combination of {*h*(−3), *α*_min_, *Δα*} turns out to be optimal compared to other combinations of parameters. We have obtained 98.4% of averaged discriminant accuracy for every two species by SVMKM with the 10 − fold cross validation, while the accuracy reaches 93.96% for the over-all 15 species. Software based on our work can be designed and coded, for that purpose, we provided the corresponding flow chart in the Figure 14.

We should point out that most of the existing work on texture image recognition focuses mainly on the standard multifractal analysis. Our work has shown that the MF-DFA is of particular practice for plant leaf identification as the MF-DFA multifractal parameters can be combined to distinguish similar but different leaf textures.
